# SYST-08. A phase II trial of concurrent Sunitinib, Temozolomide and Radiation Therapy followed by adjuvant Temozolomide for newly diagnosed Glioblastoma patients with an unmethylated MGMT gene promoter (A01-M121-11A, McG1132)

**DOI:** 10.1093/noajnl/vdab112.036

**Published:** 2021-09-21

**Authors:** Mame Daro Faye, Siham Sabri, Paula De Robles, Raman Agnihotram, Alexander Torres-Vasquez, Jacob Easaw, Bassam Abdulkarim

**Affiliations:** 1 Department of Radiation Oncology, Cedars Cancer Centre, McGill University Health Centre, Montreal, QC, Canada; 2 McGill University Heatlh Centre Research Institute, Montreal, QC, Canada; 3 Department of Pathology, Faculty of Medicine and Health Sciences, McGill University, Montreal, QC, Canada; 4 Division of Neuro-Oncology, Tom Baker Cancer Center and University of Calgary, Calgary, Alberta, Canada; 5 Division of Medical Oncology, Tom Baker Cancer Center and University of Calgary, Calgary, Alberta, Canada; 6 Department of Medicine, Division of Experimental Medicine, McGill University, Montreal, QC, Canada

## Abstract

**INTRODUCTION:**

Despite advances in treatment modalities, the overall prognosis of GBM remains dismal, particularly for patients with unmethylated MGMT promoter. Thus, alternative treatment strategies are warranted. Our group has previously shown that addition of Sunitinib (SU11248) to standard therapy significantly improved the response of unmethylated MGMT cells through decreased angiogenicity and tumorigenicity. In this phase II trial, we tested for the first time the combination of Sunitinib with RT and Temozolomide in newly diagnosed MGMT unmethylated GBM patients.

**METHODS:**

Patients with histologically confirmed WHO Grade IV GBM and MS-PCR confirmed unmethylated MGMT promoter, age 18-70, KPS ≥70, life expectancy ≥6 months were eligible. 41 patients treated between 2012 and 2017 were screened, 37 of which were eligible. Patients received 12.5 mg of daily Sunitinib for 7 days, followed by concurrent RT, Temozolomide and 12.5 mg Sunitinib for 6 weeks, then adjuvant Temozolomide x6 cycles. RT and Temozolomide doses were as per standard of care. Primary objective was PFS as assessed by RANO criteria, secondary objectives were OS and safety.

**RESULTS:**

Median follow-up time was 15 months. Median PFS was 7 months (95%CI, 6.7-7.2) and 6-month PFS was 59.3%. Median OS was 13 months (95%CI, 12.62-13.37) and 2-year OS was 17.8%. Two patients had OS >50 months, with one surviving 71 months. Having received >3 cycles of adjuvant Temozolomide, surgery at progression or age ≤65 significantly predicted for better OS, with hazard ratios of 0.184 (p=0.001), 0.402 (p=0.026) and 10.017 (for age >65, p=0.002) respectively. Grade ≥3 thrombocytopenia occurred in 18.9% of patients, grade ≥3 neutropenia in 10.8% and grade ≥3 thromboembolic events in 13.5%. There were no grade 5 evens.

**CONCLUSION:**

Addition of Sunitinib to RT and Temozolomide was well tolerated and survival outcomes compared favorably to the current standard of care for GBM patients with unmethylated MGMT promoter status.

